# Remarks on sonographic features of novel category III of the novel Bethesda System for Reporting Thyroid Cytopathology in thyroidology

**DOI:** 10.1590/1806-9282.20250537

**Published:** 2025-10-17

**Authors:** Ilker Sengul, Demet Sengul

**Affiliations:** 1 Giresun University, Faculty of Medicine, Division of Endocrine Surgery – Giresun, Turkey.; 2 Giresun University, Faculty of Medicine, Department of General Surgery – Giresun, Turkey.; 3 Giresun University, Faculty of Medicine, Department of Pathology – Giresun, Turkey.

Dear Editor,

A posteriori, indeterminate cytology and non-diagnostic outcomes in fine-needle aspiration (FNA) of the delicate papillon gland, the thyroid, remain a dynamic and challenging issue for human beings in thyroidology^1-8^. We have read with a great deal of interest the research article entitled "Ultrasonographic Characteristics of Thyroid Nodules with Nondiagnostic and Atypia of Undetermined Significance in Fine-Needle Aspiration Cytology"^9^. This utilitarian research demands investigating the potential efficacy of sonographic features of the nodules with Category I and III, The Bethesda System for Reporting Thyroid Cytopathology (TBSRTC), obtained by a 22-Gauge (G) needle without utilizing any topical and/or local anesthetic agents for clinical management^9^. For this purpose, Bozer et al.^9^ incorporated 1,252 nodules in their study during 4 years. The authors declared in their study that no significance was observed in Category I and III, in terms of their studied parameters, such as age, sex, numeric nodular value, localization, score, and sonographic features. They also emphasized no significance in the nodular diameters under and over 10 mm in size. Nevertheless, a wide range of needle sizes, 20–27-Gauge (G), have been used for FNA applications prior to cytologic evaluations in different geographic regions worldwide: 25–27-G in most of the West and 21–22-G in Japan. Herewith, would using thicker or finer needles alter this work's outcome(s)? Withal, would be performing topical and/or local anesthesia in collaboration with this interventional procedure move on the aftermath(s) of this study?^10-15^. What's more, would a feasible execution of both novel subgroups, per se, of Category III, TBSRTC, 3rd edition, convert or commute the porism of this study?^1-4,8,16^ This issue merits further investigation. We thank Bozer et al.^9^ for their valuable study in thyroidology.

## Data Availability

The datasets generated and/or analyzed during the current study are available from the corresponding author upon reasonable request.
